# PD25 - Diagnostic specificity of symptoms seen in Double Blind Placebo Control Food Challenges (DBPCFCs). Do subjective symptoms lead to false positive test results and overdiagnosis?

**DOI:** 10.1186/2045-7022-4-S1-P25

**Published:** 2014-02-28

**Authors:** Vasiliki-Lydia Sideridou, Bertine M Flokstra de Blok, Boudewijn J Kollen, Sicco van der Heide, Jeanet Kukler, Anne-Marie Oomkes-Pilon, Anton-Ewoud Dubois

**Affiliations:** 1 Department of Pediatric Pulmonology and Allergy, University Medical Centre Groningen, University of Groningen, The Netherlands

## Background

The double-blind placebo-controlled food challenge (DBPCFC) is considered to be the gold standard for diagnosing food allergy in children. During the DBPCFC, both objective and subjective symptoms are observed. Although subjective symptoms are thought to be less specific than objective symptoms, it is not known whether objective symptoms have a stronger association with a true positive result than subjective symptoms.

## Aim

The aim of this study was to analyze whether subjective symptoms are less specific than objective symptoms and whether they lead to overdiagnosis compared to objective symptoms, and also to rank symptoms according to specificity.

## Methods

Data were obtained from the food challenge unit database of University Medical Center Groningen. We included children that had a complete DBPCFC and a history of suspected food allergy. We assessed the specificity of the symptoms according to the relative risk (RR) and the 95% Confidence Interval (95%CI).

## Results

Individual symptoms showed a wide range of RR (1.777 to 0.809). The symptom with the highest RR was utricaria (RR= 1.977, 95% CI [1.857, 2.106]). However, the subjective symptom “strange taste” (RR= 1.923, 95%CI [1.720, 2.151]) was the seventh most specific symptom on the list. Certain symptoms, both subjective and objective, showed no significant association with a positive DBPCFC and were thus non-specific.

## Conclusion

Symptoms differ in their specificity as indicated by a broad range of RRs in relation to a positive test outcome. Subjective symptoms are not less specific than the objective ones, and therefore do not lead to false positive results and overdiagnosis. Protocols on interpretation of DBPCFCs should probably be based on symptom specificity rather than whether symptoms are subjective or objective.

